# Changes in device-measured daily physical activity over one year in memory clinic patients

**DOI:** 10.1186/s11556-026-00414-0

**Published:** 2026-05-07

**Authors:** Kim Frederik Gundrosen, Kristin Taraldsen, Karen Sverdrup, Anne-Brita Knapskog, Geir Selbæk, Gro Gujord Tangen

**Affiliations:** 1 Department of Rehabilitation Science and Health Technology, OsloMet – Oslo Metropolitan University, Oslo, Norway; 2 Vestfold Hospital Trust, The Norwegian National Centre for Ageing and Health, Tønsberg, Norway; 3 Department of Geriatric Medicine, Oslo University Hospital, Oslo, Norway; 4 Faculty of Medicine, University of Oslo, Oslo, Norway

**Keywords:** Physical activity, Accelerometers, Dementia, Memory clinic, Cognitive impairment

## Abstract

**Background:**

This longitudinal study explored the one-year change in device-measured daily physical activity in patients attending a memory clinic.

**Methods:**

Physical activity was recorded in 27 memory clinic patients over four days using accelerometers (activPAL3 micro) at baseline and one-year follow-up. Daily physical activity outcomes included upright time, standing time, walking time, number of steps, number of transitions, mean upright event length, and maximum upright event length. Changes between baseline and follow-up were analysed using paired sample t-tests.

**Results:**

Patients’ mean (SD) age was 69.4 (8.1) years, and 14 (51.9%) were women. The only significant change was a decrease in maximum upright event length from a mean (SD) of 84.3 (31.6) minutes at baseline to 59.2 (22.5) minutes at one-year follow-up (*p* < 0.001).

**Conclusion:**

Daily physical activity volume remained stable over one year, but the reduction in maximum upright event length indicates a shift in activity distribution.

## Introduction

Physical activity benefits people with dementia by supporting physical performance, functional independence, activities of daily living, and attenuating cognitive decline [1, 2]. Qualitative studies further describe physical activity as a source of identity, mastery, well-being, and enjoyment [3]. Despite these benefits, cross-sectional studies show that people with dementia are less physically active than age-matched, cognitively healthy controls [4, 5].

Although some longitudinal studies have explored the relationship between physical activity and cognition, they have focused on changes in cognitive function [6, 7]. A review of accelerometer-based habitual physical activity in dementia identified a lack of longitudinal studies and considerable variation in methods and reported metrics, highlighting the need for clearer evidence on how activity patterns change over time [8]. The cross-sectional data included in the review indicated that people with mild cognitive impairment (MCI) showed differences in patterns and variability of daytime physical activity, rather than in overall activity volume, compared with controls [8]. Understanding longitudinal changes in physical activity may be relevant in memory clinic populations, as changes in daily mobility may both reflect and contribute to emerging functional decline in a potentially bidirectional relationship. The review also noted uncertainty about whether different stages of cognitive impairment show distinct activity behaviours due to limited data [8]. Device-measured physical activity enables a detailed assessment of daily movement, offering insight into both total activity volume and how movement is accumulated across the day, such as through upright event length and transitions [9]. This study aimed to examine one-year changes in seven daily physical activity outcomes in memory clinic patients with cognitive impairment and dementia.

## Methods

We recruited participants at the Memory Clinic at Oslo University Hospital in Oslo, Norway, who were included in the Norwegian Registry of Persons Assessed for Cognitive Symptoms (NorCog) [10], and who were able to walk independently without walking aids. Exclusion criteria included the inability to complete cognitive tests without a translator. While the cross-sectional study is still ongoing, this brief report focuses on the completed longitudinal part of the study, which involved baseline testing and a planned one-year follow-up starting in January 2017. The longitudinal part ended prematurely in March 2020 due to COVID-19 restrictions, resulting in 75 participants being lost to follow-up. There were no significant differences between these participants and the 27 with follow-up data in age, sex, gait speed, or MMSE-NR3 scores (all *p* > 0.05).

### Demographic and clinical variables

Age (years), sex (male/female), global cognitive function, and gait speed were recorded at baseline. The International Classification of Diseases, version 10 (ICD-10) was used for diagnoses of MCI and dementia [10], while subjective cognitive decline (SCD) was applied when criteria for MCI or dementia were not met. To describe global cognitive function, we used the third Norwegian revision of the Mini Mental State Examination (MMSE-NR3), scored 0–30 points, higher scores indicating better performance [11]. Usual gait speed (meters per second) was determined using the fastest of two recorded 4-meter walk tests from the Short Physical Performance Battery [12].

### Physical activity outcomes

Daily physical activity was obtained using the activPAL3 micro (PAL Technologies Ltd, Glasgow, UK), a triaxial accelerometer attached to the participant’s thigh with waterproof tape and worn continuously for four days at baseline and follow-up. We aimed for four valid 24-hour days, supported by evidence that three to four days provide reliable activity estimates in older adults [13], and to balance participant burden. Wear time ranged from one to four valid days at both timepoints: one participant had a single valid day, two had three days, and the rest had four days. Sensitivity analyses excluding those with only one day did not alter the results. All files underwent manual quality checks. Using the PAL Software Suite (version 8) and a custom-made spreadsheet provided by PAL Technologies Ltd, we derived seven daily physical activity outcomes relevant to overall mobility and health: Upright time (minutes in standing and walking), standing time (minutes), walking time (minutes), number of steps, number of sit-to-stand transitions, and mean and maximum upright event length (minutes). ActivPALs have generally shown valid measures of both transitions, upright time, standing time, and sedentary time [14] and step count [15] in older adults.

### Statistical analysis

Participants’ characteristics were described using descriptive statistics. One-year change in physical activity was analysed using paired samples t-tests or Wilcoxon signed rank test. For physical activity variables that showed significant change, we performed multiple linear regression analyses to explore if age, MMSE-NR3, or gait speed at baseline were associated with change. We also conducted additional descriptive analyses and exploratory group comparisons to contextualise observed changes. The significance level was set at *p* < 0.05. All data analysis was performed using IBM SPSS Statistics 27©.

## Results

**Fig. 1:**
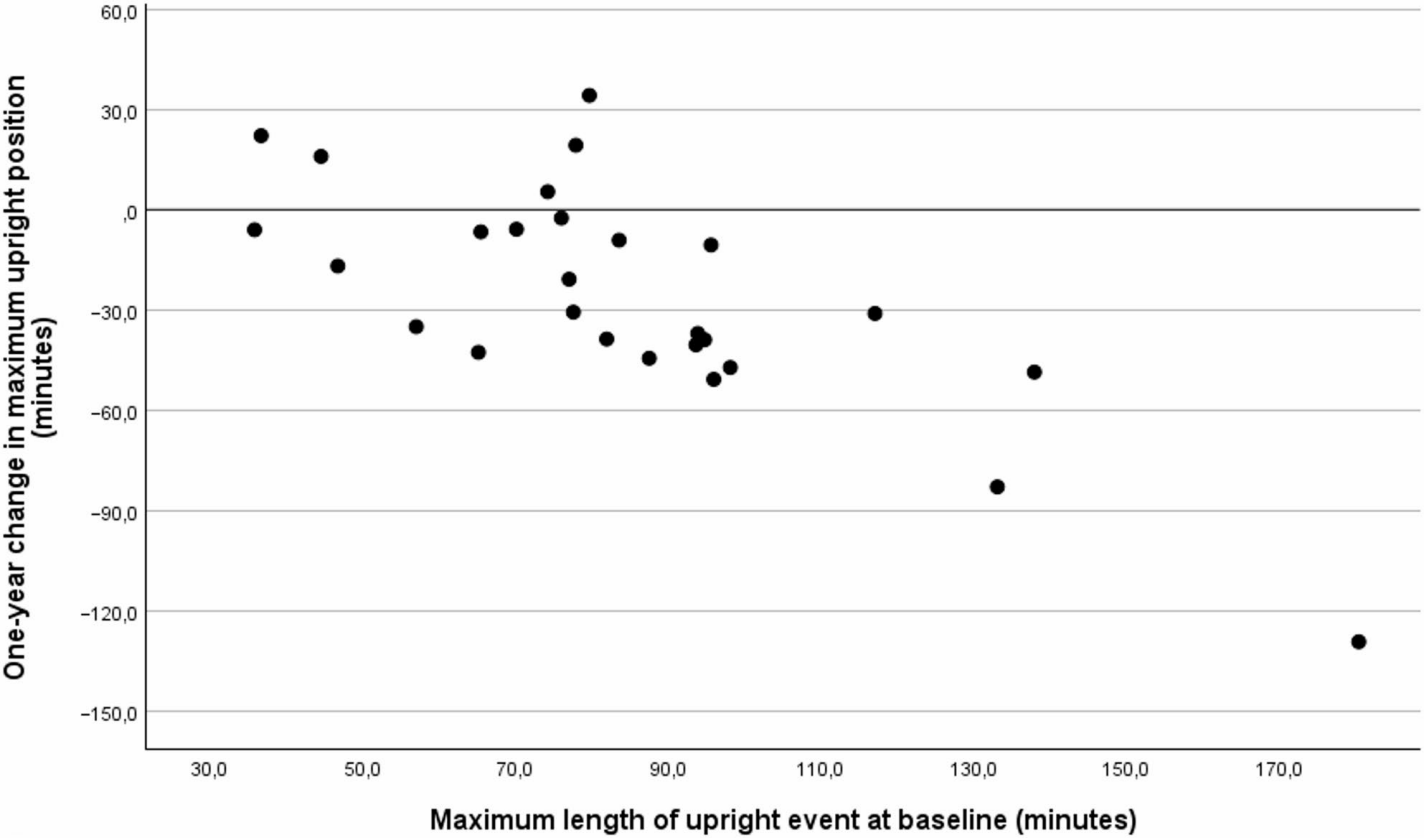
Differences in minutes of maximum upright event length between baseline and one-year follow-up plotted against the baseline results. The horizontal line indicates no difference between the two time points

We included 27 participants (14 women) with complete physical activity data at baseline and one-year follow-up. At baseline, mean (SD) age was 69.4 (8.1) years, median (IQR) MMSE-NR3 score was 25 (8), and mean (SD) gait speed was 1.0 m/s (0.2 m/s). Seventeen participants had dementia, nine had MCI, and one was categorised with SCD. The participants’ daily averages (SD) at baseline were 360.1 (126.3) minutes in upright time (262.6 min standing and 97.5 min walking), 7874.0 (2913.9) steps, and 53.0 (13.9) transitions. Mean upright event length was on average 6.9 (2.6) minutes, and maximum upright event length was on average 84.3 (31.6) minutes. Follow-up occurred after a mean (SD) of 383.4 (42.6) days (range 307–463). Maximum upright event length decreased from mean (SD) 84.3 (31.6) minutes at baseline to 59.2 (22.5) minutes at follow-up (mean difference 25.1 (33.9) minutes, *p* < 0.001; Cohen’s d = 0.74, 95% CI [0.31, 1.16]) (Fig. 1). None of the other physical activity measures showed statistically significant changes (all p’s > 0.05) (Table 1). At baseline, 18.5% of participants had a maximum upright event length shorter than 60 min, increasing to 64.3% at follow-up. Figure 1 displays the one-year change in maximum upright event length for all individual participants, sorted by their baseline maximum length in an upright event.

Change in maximum upright event length from baseline to follow-up was not explained by age (*p* = 0.73), MMSE-NR3 score (*p* = 0.71), or gait speed (*p* = 0.25). In an exploratory comparison, participants with SCD/MCI showed a larger decline in maximum upright event length than those with dementia: mean (SD) difference − 36.9 (21.7) sec vs. -18.3 (38.3) sec, although statistically non-significant (*p* = 0.066).

**Table 1:** Change in daily physical activity from baseline to one-year follow-up

Variables, mean (SD)	Baseline	1 year follow-up	Mean difference (95% CI)	*p*
Upright time, minutes	360.1 (126.3)	367.7 (138.5)	7.7 (-23.4, 38.7)	.62^a^
Standing time, minutes	262.6 (108.3)	269.5 (113.1)	6.9 (-17.5, 31.3)	.57^a^
Walking time, minutes	97.5 (35.2)	98.3 (35.5)	0.8 (-14.3, 15.8)	.92^a^
Steps, number	7874 (2914)	7920 (3064)	46 (-1224, 1317)	.83^b^
Transitions, number	53 (14)	60 (30)	7(-3, 18)	.10^b^
Mean upright event length, minutes	6.9 (2.6)	6.6 (3.3)	-0.3 (-1.2, 0.7)	.40^b^
Maximum upright event length, minutes	84.3 (31.6)	59.2 (22.5)	-25.1 (-38.6, -11.7)	< 0.001

^a^Paired samples t-test

^b^Wilcoxon Signed Ranks Test

## Discussion

In this longitudinal study of 27 participants with SCD, MCI, or dementia, we examined changes in daily physical activity measured by accelerometers at two time points over one year. Of the seven daily physical activity outcomes, six remained stable, while a significant decline was observed in maximum upright event length. This finding suggests a shift in how physical activity is accumulated, from longer continuous periods to shorter, more frequent periods. These results corroborate and expand the results from the cross-sectional studies in the review by Mc Ardle et al. [8]. Potential explanations remain hypothetical, but such a change may reflect adaptations to cognitive challenges, for example, reduced spatial orientation leading individuals to stay closer to home, or physical limitations such as reduced stamina or balance. Alternative contributing factors cannot be ruled out. Day‑to‑day behavioural variation, like running errands or random social activities, or contextual factors including changes in caregiver routines, may also affect how activity is accumulated. Although the number of transitions showed a slight increase, this change was not statistically significant, which may be explained by the lack of statistical power. These results indicate stable overall activity volume may camouflage potentially important changes in activity behaviour. Future research should evaluate whether such changes can serve as early indicators of declining mobility and also explore which factors are associated with these changes.

A study of Alzheimer’s disease progression in memory clinics found that almost half of the patients showed slow or undetectable progression over two years [16], suggesting that the one-year follow-up may be too short to detect major changes in physical activity in early dementia. Still, early changes may be observable. In our exploratory comparison, participants with SCD/MCI showed a greater decline in maximum upright event length than those with dementia, suggesting that alterations in activity accumulation may begin before a formal diagnosis. However, this finding should be interpreted cautiously, given the small subgroup sizes.

This study has several limitations. The sample was small and heterogeneous in cognitive status and underlying dementia etiology, which warrants caution when interpreting the findings and limits in-depth exploration of additional activPAL metrics, such as sedentary behaviour, as well as disease-specific trajectories. A major strength, however, is the longitudinal design, which adds valuable data on physical activity in memory clinic patients. To our knowledge, this is the first longitudinal study to examine changes in device-measured physical activity in this population.

## Conclusion

In this longitudinal study of memory clinic patients, most daily physical activity outcomes remained stable over one year. A decline in maximum upright event length was observed, with a moderate effect size, suggesting a possible shift in how activity was accumulated. Given the small sample, these findings should be interpreted cautiously. Studies with larger samples and longer follow‑up are needed to confirm and expand these findings.

## Data Availability

The dataset can be made available by reasonable request to the last author. However, data sharing was not included in the original ethical approval and must therefore be applied for and approved before any data can be shared.

## Abbreviations

NorCog: Norwegian Registry of Persons Assessed for Cognitive Symptoms
ICD-10: The International Classification of Diseases, version 10
MCI: Mild cognitive impairment
SCD: Subjective cognitive decline
MMSE-NR3: Third Norwegian revision of the Mini Mental State Examination
